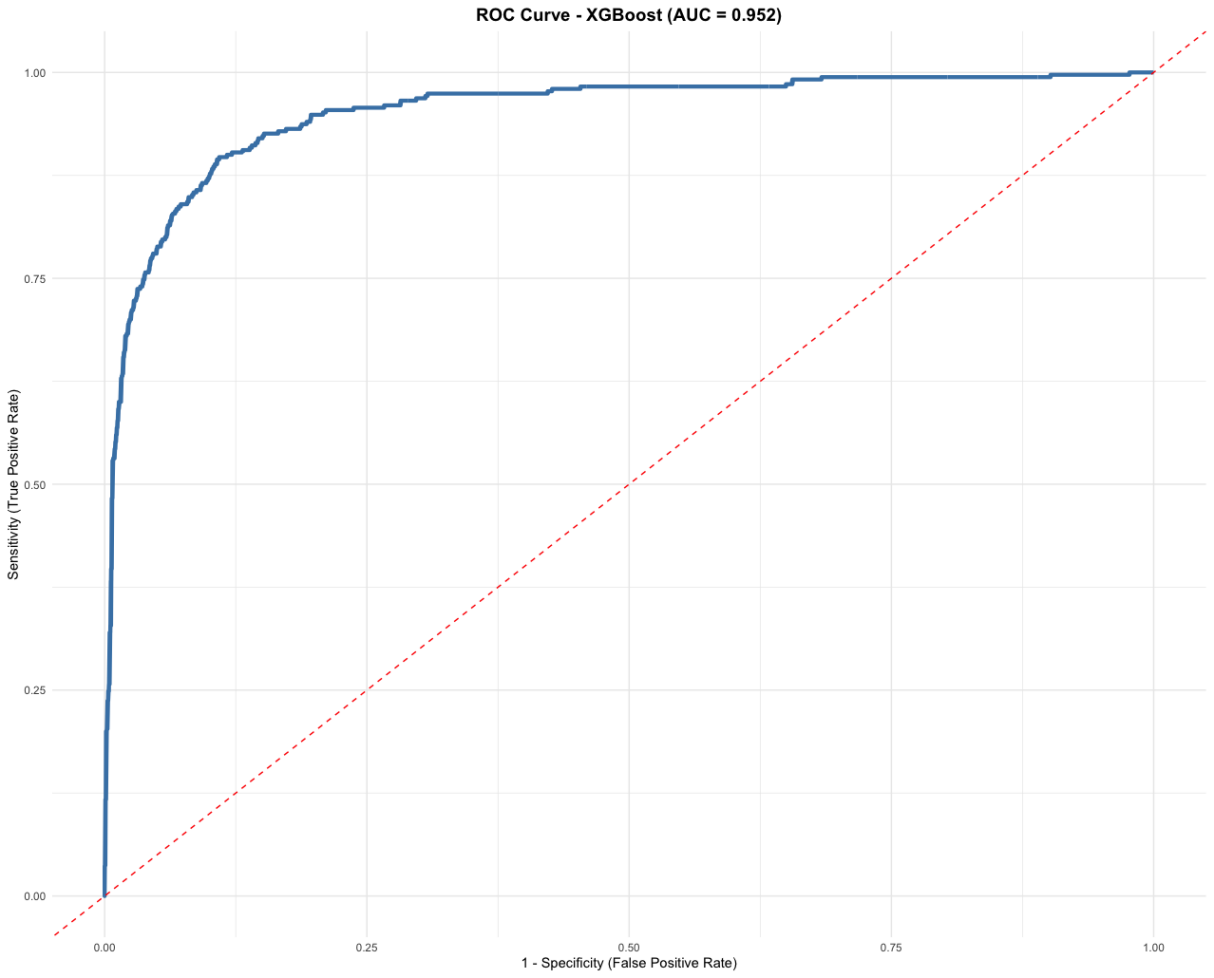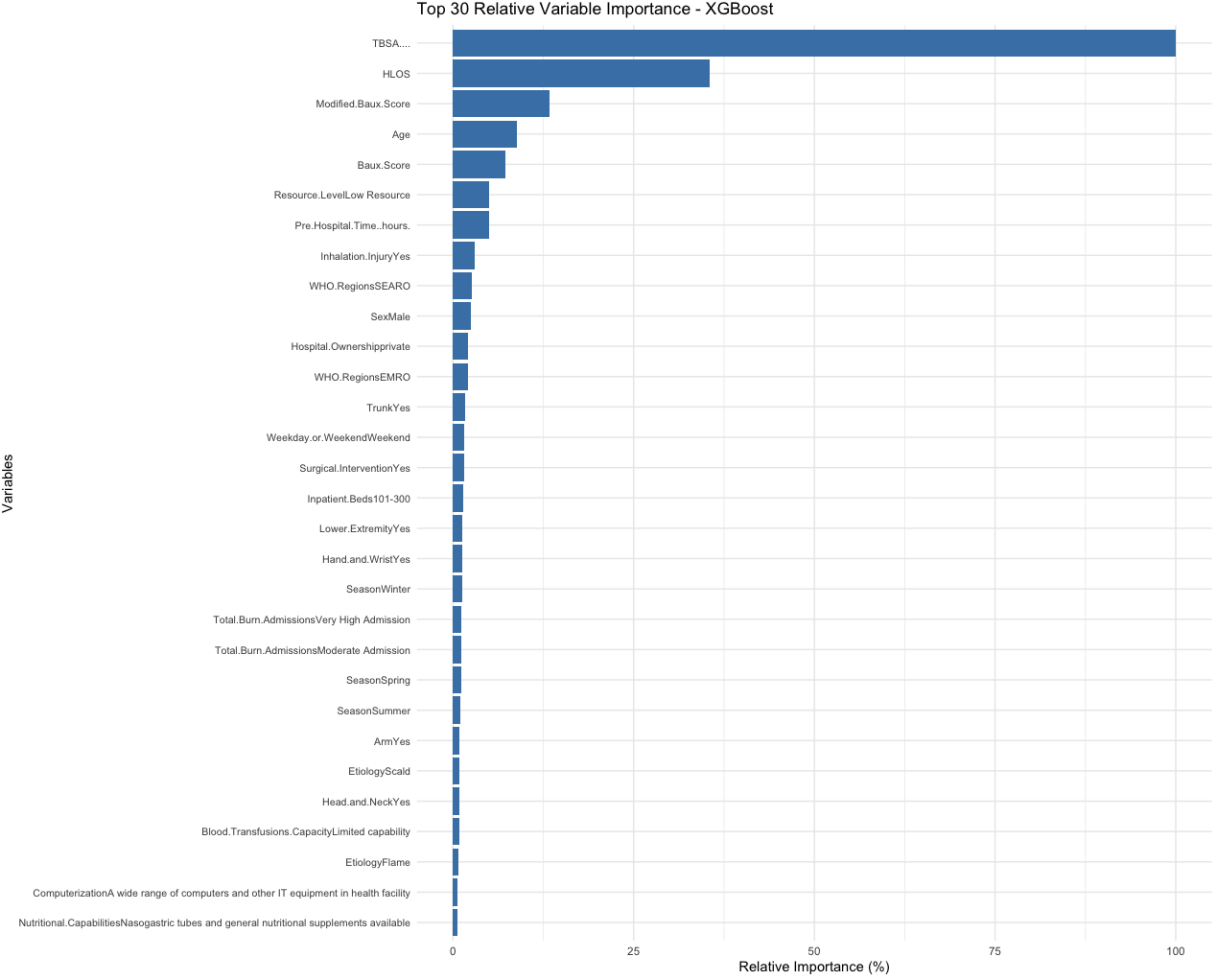# 14 Ensemble Machine Learning Models for Burn Mortality Prediction Using the WHO Global Burn Registry

**DOI:** 10.1093/jbcr/iraf019.014

**Published:** 2025-04-01

**Authors:** Daniel Najafali, Megan Najafali, Logan Galbraith, Hilary Liu, Michael Pozin, Erik Reiche, Raman Mehrzad, Quincy Tran, Sameer Patel, Victor Stams, Francesco Egro

**Affiliations:** Carle Illinois College of Medicine; Loyola University Chicago Stritch School of Medicine; Northeast Ohio Medical University; University of Pittsburgh Medical Center; Carle Illinois College of Medicine; University of Pittsburgh Medical Center; Ocean Plastic Surgery Center; University of Maryland School of Medicine; Fox Chase Cancer Center; Carle Foundation Hospital; University of Pittsburgh Medical Center

## Abstract

**Introduction:**

Burn injuries represent a significant global health challenge, with mortality prediction being a critical component that can dictate patient care and resource allocation. This study aims to apply ensemble machine learning models to predict burn mortality using a large database that captures burns from diverse global settings. We identified the most important predictors of burn mortality.

**Methods:**

The dataset comprised patient records from the WHO Global Burn Registry from its inception. The primary outcome of interest was mortality after burn injury. We used random forest (RF), gradient boosting machine (GBM), and extreme gradient boosting (XGBoost) to develop predictive models. Their performance was assessed based on accuracy, area under the curve (AUC), F1 score, sensitivity, specificity, and balanced accuracy. Variable importance was also analyzed to determine the most influential factors in predicting burn mortality.

**Results:**

The models were evaluated using data from 9,130 burn patients and 35 predictors. The RF model achieved an accuracy of 91.84% (95%CI: 90.48–93.05%), an AUC of 0.843, and an F1 score of 0.9503, with a sensitivity of 96.54% and specificity of 72.00%. The GBM model yielded an accuracy of 91.73% (95%CI: 90.37–92.95%), an AUC of 0.9503, and an F1 score of 0.9497, with a sensitivity of 96.61% and specificity of 71.14%. The XGBoost model demonstrated the best overall performance, with an accuracy of 91.95% (95%CI: 90.60–93.15%), an AUC of 0.9518, and an F1 score of 0.9505, along with a sensitivity of 95.73% and the highest specificity at 76.00%. The balanced accuracy for XGBoost was 85.86%, outperforming both the RF and GBM. The top 5 predictors of mortality for the XGBoost model were: 1) TBSA (%), 2) length of stay (days), 3) Baux score, 4) age, and 5) management in a low-resource setting.

**Conclusions:**

All three ensemble learning models—RF, GBM, and XGBoost—demonstrated strong predictive ability for burn mortality on a global scale, with XGBoost performing the best across all metrics. These findings suggest that machine learning models, especially XGBoost, can serve as valuable tools for burn providers in predicting outcomes across resource levels. The development of a risk stratification tool based on these models could potentially optimize resource allocation for burns.

**Applicability of Research to Practice:**

This study captures the potential of ensemble machine learning models to enhance predictive accuracy in burn mortality outcomes using real-world data from the World Health Organization. These models can hopefully assist in triaging burn patients, optimizing resource allocation, and ultimately improving decision-making in critical care settings in high- and low-resource regions.

**Funding for the Study:**

N/A